# A framework for the molecular identification of CHIP for clinical research

**DOI:** 10.1016/j.xhgg.2026.100575

**Published:** 2026-01-20

**Authors:** Philip Harraka, Robert L. O’Reilly, Jared Burke, Paul Yeh, Kerryn Howlett, Kiarash Behrouzfar, Daniele Belluoccio, Amanda Rewse, Brigid M. Lynch, Kristen J. Bubb, Stephen J. Nicholls, Roger L. Milne, Melissa C. Southey

**Affiliations:** 1 Precision Medicine, School of Clinical Sciences at Monash Health, Monash University, Clayton, VIC 3168, Australia; 2 Monash Haematology, Monash Health, Clayton, VIC 3168, Australia; 3 Department of Medicine, School of Clinical Sciences at Monash Health, Monash University, Clayton, VIC 3168, Australia; 4 Diagnostics and Genomics Group, Agilent Technologies Australia Pty Ltd., Mulgrave, VIC 3170, Australia; 5 Clinical Genomics, School of Translational Medicine, Monash University, Melbourne, VIC 3004, Australia; 6 Cancer Epidemiology Division, Cancer Council Victoria, East Melbourne, VIC 3002, Australia; 7 Centre for Epidemiology and Biostatistics, Melbourne School of Population and Global Health, The University of Melbourne, Parkville, VIC 3010, Australia; 8 Biomedicine Discovery Institute, Monash University, Clayton, VIC 3168, Australia; 9 Victorian Heart Institute, Monash University, Clayton, VIC 3168, Australia; 10 Victorian Heart Hospital, Clayton, VIC 3168, Australia

**Keywords:** clonal hematopoiesis, clonal hematopoiesis of indeterminate potential, CHIP, genetics, framework

## Abstract

Clonal hematopoiesis of indeterminate potential (CHIP) is associated with many diseases of aging. Large research initiatives are needed to develop clinical guidelines for the management of individuals with CHIP and their risk of disease. However, little guidance is available for the classification of variants as CHIP associated or how to identify individuals consistently and systematically as having CHIP. This study aimed to develop and execute a resource-mindful framework for identifying individuals with CHIP, and those without, for downstream clinical studies. This framework was used to categorize CHIP in a cross-section of 2,328 participants from the Australian Breakthrough Cancer Study. DNA extracted from saliva samples was sequenced for a panel of ten gene regions that frequently carry variants that are associated with CHIP. Variants in these regions were curated for CHIP according to field-specific criteria. Individuals were categorized as either CHIP positive, CHIP negative, or CHIP indeterminate based on their variant findings. Sequencing was successfully performed on 2,328 individuals. The mean age (± standard deviation) was 68 ± 3 years, and 48% were men. 347 participants (15%) were identified as CHIP positive with a total of 400 CHIP-associated variants. 1,442 participants (62%) were considered CHIP negative based on finding no somatic variation within the target regions. The remaining 539 (23%) were considered CHIP indeterminate because they had at least one variant that could not be interpreted. This framework provides a consistent approach to the categorization of individuals as CHIP positive or CHIP negative for clinical research and provides an opportunity for improved harmonization in the curation of CHIP.

## Teaser

This study developed a framework to support the identification of individuals with clonal hematopoiesis of indeterminate potential (CHIP), and those without, for recruitment into a clinical study. 2,328 individuals aged 64–74 were screened, and 15% were identified to have CHIP, 62% did not have CHIP, and 23% had indeterminate findings.

## Introduction

Clonal hematopoiesis of indeterminate potential (CHIP) presents a global health challenge and opportunity for prevention due to its association with many diseases of aging. CHIP is associated with a 4-fold increased risk of hematologic cancer and a 75% increased risk of coronary heart disease.^1^ Currently, clinical studies are measuring the cardiovascular risk of CHIP including plaque burden, composition, and progression, and their relationship with established disease biomarkers. Clonal hematopoiesis risk prediction models are also being developed such as the clonal hematopoiesis risk score (CHRS), which can predict which individuals with CHIP are at the highest risk of a myeloid neoplasm within 10 years,^2^ and MN-predict, which predicts the likelihood of developing a myeloid neoplasm within 15 years.^3^ However, clinical research is complicated by the difficulty of consistently and systematically identifying individuals with CHIP and those without.

CHIP is a form of clonal hematopoiesis, whereby progeny cells harbor somatic mutations in myeloid malignancy driver genes that are detected in blood- or bone-marrow-derived DNA at a variant allele fraction (VAF) ≥0.02 in individuals without a diagnosed hematologic disorder or unexplained cytopenia.^4^ However, this definition provides little practical guidance on what genes to consider, how to classify CHIP based on the variants detected and their VAF, or how to identify individuals who are CHIP negative with high confidence.

This study presents a framework for identifying individuals with CHIP, and those without, for recruitment into clinical and epidemiological research. This framework was utilized to investigate CHIP in 2,425 individuals sampled from a contemporary Australian cohort study. Saliva-derived DNA was sequenced for CHIP because it was available from all participants and because saliva was previously demonstrated to be an appropriate DNA source to detect CHIP-associated variants,^5^ with the VAF detected in saliva-derived DNA shown to be highly concordant with the VAF detected in buffy-coat-derived DNA (*R*^2^ = 0.95).^6^ Ten gene regions were selected for investigation (*DNMT3A*, *TET2*, *ASXL1*, *PPM1D*, *TP53*, *SRSF2*, *JAK2*, *SF3B1*, *GNB1*, and *NF1*) because they had previously been reported to carry the highest number of variants associated with CHIP in the UK Biobank, the largest known investigation of CHIP.^7^ This work details the approach used to filter and interpret variation in these genes, the prevalence of recognizable CHIP, and the variants observed.

## Material and methods

### Study design and participant selection

A total of 2,425 participants were selected from the Australian Breakthrough Cancer (ABC) Study, a cohort of 56,282 Australian residents who reported no personal history of cancer at recruitment in 2014–2018, when aged 40–74 years. The aim of the ABC Study is to investigate the causes of cancer and other non-communicable diseases. Individuals were eligible for the present study if they (1) provided a saliva sample at recruitment, (2) were aged 64 years or older at the time of sample collection, (3) reported no personal history of heart attack or stroke at recruitment, and (4) were living in Victoria at study selection (2023). The ABC Study is approved by the Cancer Council Victoria Human Research Ethics Committee (#1403). ABC Study participants provided informed consent by logging into a secure web portal and completing a date- and time-stamped tick box.

### Data and biological sample collection

Participants were asked to complete epidemiological questionnaires that included sections on demographic characteristics and cancer-related risk factors including smoking history. Participants were invited to provide a saliva sample using the Oragene OG-500 kit (DNAGenotek), which was sent to participants and returned to Precision Medicine, Monash University via the postal service.

### Sample handling and bioinformatics processing

DNA extraction, panel design, sequencing, and bioinformatics pipelines were validated to detect variants at low VAF and previously published^5^^,^^8^ (with minor adaptation here to include a second variant caller). In brief, DNA was extracted from saliva samples using either the Qiagen Symphony or Chemagic platforms. Library preparation of samples was conducted using Agilent’s SureSelect XT HS2 protocol. Probe-based capture targeted the exons and proximal intron-exon junctions of a single large panel designed to have non-uniformity of sequencing depth for different genes. This panel included the ten gene regions that were of interest to this study (Table S1) targeted at a read depth of at least 500×.^8^ Massively parallel sequencing was performed using the NextSeq 550 (Illumina). A custom Nextflow pipeline mapped the raw sequencing files to the GRCh38 human reference genome, called variants using both VarDict (v.1.8.3) and Mutect2 (v.4.4.0), and annotated the variants using Ensembl-VEP (v.111). A DNA sample was considered successfully sequenced when at least 80% of the targeted panel was covered at a read depth of at least 50× (depth cutoff was selected based on the nature of the non-uniformity of the designed panel^8^). Further filtering on variant-specific sequencing quality metrics for CHIP analysis is described below. All variant filtering was performed using PostgreSQL (v.12.18).

### Variant origin: Somatic or germline

The study framework used the VAF to infer whether the variant identified in the saliva-derived DNA was somatic or germline in origin. Due to the expectation that germline variants would have a VAF greater than 0.25, all variants with a VAF ≤0.25 were considered likely somatic. A variant with a VAF >0.25 was suspected to be germline unless it was a CHIP-associated variant.

### Variant filtering

**Figure 1:**
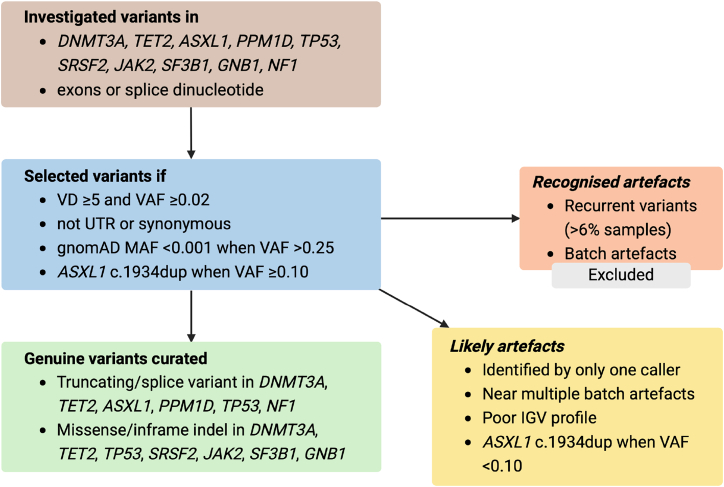
Workflow illustrating how variants were filtered IGV, Integrative Genomics Viewer; indel, insertion-deletion; MAF, minor allele frequency; UTR, untranslated region; VAF, variant allele fraction; VD, variant depth.

Variant filtering (Figure 1) was performed on variants from the protein-coding exons of *DNMT3A* (MIM: 602769) (GenBank: NM_022552.5), *TET2* (MIM: 612839) (GenBank: NM_001127208.3), *ASXL1* (MIM: 612990) (GenBank: NM_015338.6), *PPM1D* (MIM: 605100) (GenBank: NM_003620.4), *TP53* (MIM: 191170) (GenBank: NM_000546.6), and *SRSF2* (MIM: 600813) (GenBank: NM_001195427.2), and key exons of *JAK2* (MIM: 147796) (GenBank: NM_004972.4), *SF3B1* (MIM: 605590) (GenBank: NM_012433.4), *GNB1* (MIM: 139380) (GenBank: NM_002074.5), and *NF1* (MIM: 613113) (GenBank: NM_001042492.3) that had been reported to be associated with CHIP^7^^,^^9^ (Table S1). Variant calls were filtered for those that (1) had a variant depth^7^ of ≥5 and a VAF ≥0.02, (2) were in the targeted exons or flanking splice dinucleotides, and (3) were not untranslated region (UTR) or synonymous variants. Variant calls with a VAF >0.25 were additionally filtered for those that had a minor allele frequency <0.001, or were absent, in gnomAD exomes (v.2.1.1) and genomes (v.3.1.2). This cutoff was selected to exclude common population germline variation while still retaining common CHIP-associated somatic variants that are reported in gnomAD with a minor allele frequency >0.0001, such as *DNMT3A* c.2644C>T (p.Arg882Cys), *DNMT3A* c.2645G>A (p.Arg882His), *ASXL1* c.1934dup (p.Gly646TrpfsTer12), and *JAK2* c.1849G>T (p.Val617Phe).

### Variant analysis

To be considered genuine, variants had to have been identified by both callers (where at least one of the calls reported a VAF ≥0.02 and variant depth ≥5). Instances where VarDict and Mutect2 called different variants at the same genomic location were inspected in Integrative Genomics Viewer (IGV), and, if considered genuine, the appropriate variant call and annotation was determined (Table S2). Variants were also inspected in IGV if they were (1) large insertion-deletion (indel) events (defined here as an insertion or deletion greater than 20 bp) or (2) in the *ASXL1* homopolymer located at c.1927_1934. Regarding the common CHIP-associated *ASXL1* variant c.1934dup (p.Gly646TrpfsTer12), due to the known occurrence of sequencing artifacts near this homopolymer, the variant was only considered genuine if at least one of the two calls reported a VAF ≥0.10 (cutoff described in Vlasschaert et al.^7^).

### Variant artifacts

Two types of artifacts were defined and processed independently. Recognized artifacts included (1) recurrent variants observed with a VAF between 0.02 and 0.25 in more than 6% of samples^10^ and therefore considered too common to be CHIP associated, and (2) sequencing run batch artifacts, which were variants that clustered in samples according to sequencing run; both of which were excluded. Likely artifacts included instances where the variant was (1) not identified by both callers, (2) found in an exon where multiple batch artifacts had been found (or its flanking splice dinucleotides), (3) inspected in IGV and not considered genuine (for instance, some large indels were only observed at the beginning or end of reads, or in regions of low genomic complexity), or (4) was *ASXL1* variant c.1934dup with a VAF <0.10. Likely artifacts were distinguished from recognized artifacts because they could still be genuine somatic variants.

### Determining participant CHIP status

**Figure 2:**
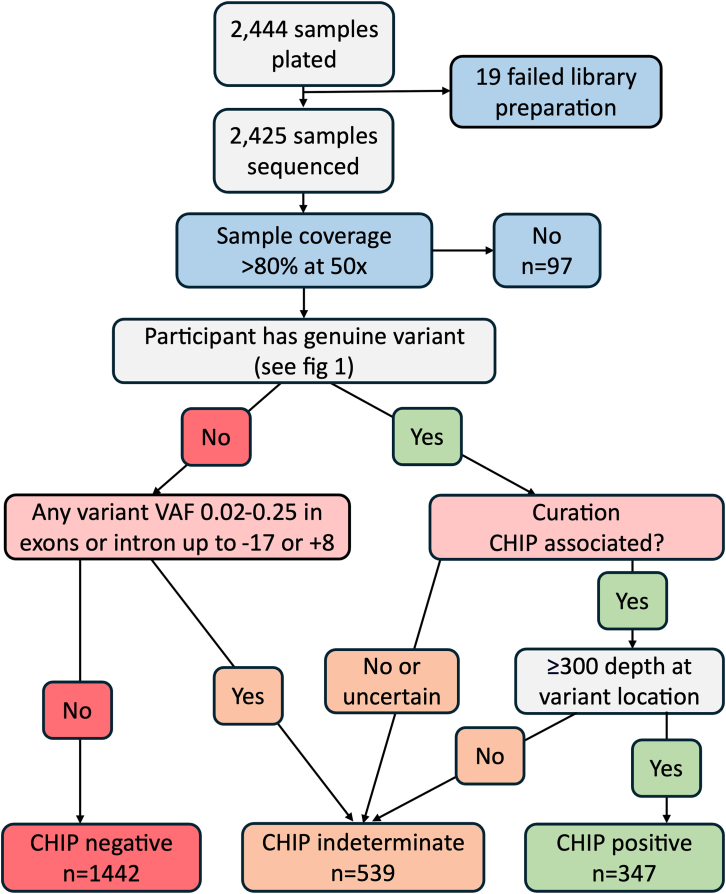
Workflow illustrating the process of determining a participant’s CHIP status

Participant CHIP status was considered either positive, negative, or indeterminate (Figure 2). Details of how variants were classified as CHIP associated are described in the results. Participants were CHIP positive if they had at least one CHIP-associated variant, and at least one of the two calls for that variant (VarDict or Mutect2) specified a read depth ≥300. This read depth cutoff was selected to reduce the number of variants that may be false positives. Participants were CHIP negative if they (1) did not carry a CHIP-associated variant (VAF≥0.02) and (2) did not carry any somatic variation (VAF ≥0.02 and ≤0.25; variant depth ≥5) within the sequenced exons or splice regions (up to −17 or +8 of intron) in the ten investigated genes. Here, the splice region is investigated (not just the dinucleotide) because variants anywhere within the splice region have the potential to impact splicing and be CHIP associated. However, variants outside the splice dinucleotide were not assessed for CHIP association because curation of these variants is more intricate, likely less precise, and often lacking in supporting evidence. Similarly, the definition of “CHIP negative” required a participant to have no UTR or synonymous variants, but these were not assessed for CHIP association. Recognized artifacts were excluded prior to determining whether participants were CHIP negative (since these are not genuine variants). However, participants who had a likely artifact were considered CHIP indeterminate (not CHIP negative) because these could still be genuine CHIP-associated somatic variants. Participant CHIP status was considered indeterminate if they (1) had a somatic variant within the exons or splice regions (up to −17 or +8) that could not be interpreted (which may include likely artifacts but not recognized artifacts) or (2) had a CHIP-associated variant but with read depth for the variant <300.

### Statistical analyses

Associations of CHIP status with age at sample collection were assessed by comparing proportions by tertiles of age and testing trends by fitting a logistic regression model including age as a continuous variable. A *p* value of <0.05 was considered statistically significant.

## Results

Sequencing was performed on 2,425 participants selected from the ABC Study. Of these, 2,328 were successfully sequenced with an average of 96% of the targeted regions covered at a minimum of 50×. The mean age (± standard deviation) was 68 ± 3 years, and 48% were men (*n* = 1,125) (Table 1). A history of smoking (defined as an average of at least 7 cigarettes per week for at least 12 months) was reported by 46% of individuals (*n* = 1,071), and 2% were current smokers at recruitment (*n* = 57).

**Table 1:** Participant demographic characteristics by CHIP status

Demographics	CHIP positive	CHIP negative	CHIP indeterminate
Participants, no. (%)	347/2,328 (15)	1,442/2,328 (62)	539/2,328 (23)
Sex, no. (%)			
Male	179/1,126 (16)	689/1,126 (61)	258/1,126 (23)
Female	168/1,202 (14)	753/1,202 (63)	281/1,202 (23)
Age in years, mean (SD)	68.6 (2.9)	68.0 (2.8)	68.2 (2.9)
Smoking status, no. (%)			
Never smoked	177/1,251 (14)	773/1,251 (62)	301/1,251 (24)
Ever smoked^a^	169/1,071 (16)	664/1,071 (62)	238/1,071 (22)
Current smoker	9/57 (16)	39/57 (68)	9/57 (16)
Past smoker	158/1,005 (16)	622/1,005 (62)	225/1,005 (22)
Not answered	2/9 (22)	3/9 (33)	4/9 (44)
Don’t know	1/6 (17)	5/6 (83)	0

CHIP, clonal hematopoiesis of indeterminate potential; no., number; SD, standard deviation.

a Individuals who reported having smoked regularly for at least 1 year were asked if they were current regular smokers.

### Variant classification

The method of classifying variants as CHIP-associated varied by gene and variant consequence (truncating, missense, or splicing) and is summarized in Table 2. In brief, truncating and splice donor or acceptor dinucleotide variants were only investigated for genes where truncating variants had been previously reported to be deleterious (*DNMT3A*, *TET2*, *ASXL1*, *PPM1D*, *TP53*, and *NF1*), and missense (or in-frame indel) variants were only investigated for genes that are known to be oncogenes or tumor suppressors in hematopoietic malignancies (*DNMT3A*, *TET2*, *TP53*, *SRSF2*, *JAK2*, *SF3B1*, and *GNB1*). Truncating variants comprised both frameshift and nonsense variants that introduced a premature termination codon (PTC). Only one frameshift variant was predicted to result in protein extension and was not considered CHIP associated because it did not impact a protein domain. Truncating variants were only considered CHIP associated if they were predicted to induce nonsense-mediated decay (NMD)^11^ or if they introduced a PTC within a functional protein domain or upstream of a different truncating variant that had been previously reported with germline disease for that gene. For a gene-specific explanation of how truncating variants were considered CHIP associated, see the gene subheadings in the supplemental results. Splice variants were only considered CHIP associated if they had been reported as pathogenic or likely pathogenic with a review status of at least “2 stars” in ClinVar (accessed June 2024) and had a spliceAI score^12^ of ≥0.20. Missense (or in-frame indel) variants were considered CHIP associated if they had been reported in a published summary of candidate missense and indel variants^7^ or, for *TET2*, were located within a domain (amino acids 1,129–1,481 or 1,843–1,936).^13^ Variant findings for each gene are described in the supplemental results. The distribution of variants identified in *DNMT3A*, *TET2*, *ASXL1*, *PPM1D*, and *TP53* are depicted using circular lollipop plots in Figure S1. A summary of the number of variants identified per gene, their consequence, and CHIP association is given in Table 3.

**Table 2:** Approach to classifying variants as CHIP associated according to gene and variant consequence

Gene	Truncating variant	Missense variant	Splice ±1,2 variant
DNMT3A	PTC upstream of last 50 bp of second-last exon	reported by Vlasschaert et al.^7^	PLP ≥2 stars in ClinVar and spliceAI ≥0.20
TET2	PTC upstream of p.Gly1936	at p.1129_1481 or p.1843_1936	PLP ≥2 stars in ClinVar and spliceAI ≥0.20
ASXL1	PTC in last 2 exons, upstream of p.Arg1415∗	N/A	PLP ≥2 stars in ClinVar and spliceAI ≥0.20
PPM1D	PTC in last 2 exons, upstream of p.Arg552∗	N/A	PLP ≥2 stars in ClinVar and spliceAI ≥0.20
TP53	PTC upstream of last 50 bp of second-last exon	reported by Vlasschaert et al.^7^	PLP ≥2 stars in ClinVar and spliceAI ≥0.20
SRSF2, JAK2, SF3B1, GNB1	N/A	reported by Vlasschaert et al.^7^	N/A
NF1	PTC upstream of last 50 bp of second-last exon	N/A	PLP ≥2 stars in ClinVar and spliceAI ≥0.20

N/A, not applicable variant consequence for that gene; PLP, pathogenic or likely pathogenic; PTC, premature termination codon.

**Table 3:** Number of variants identified according to gene, variant consequence, and CHIP association

Gene	Total variants	Truncating variants	Missense (inf indel) variants	Splice ±1,2 variants	CHIP-associated variants (DP ≥ 300)
DNMT3A	294	78	172	44	157
TET2	220	109	105	6	157
ASXL1	33	33	N/A	0	33
PPM1D	22	22	N/A	0	18
TP53	20	0	19	1	7
SRSF2	13	N/A	13	N/A	5
JAK2	14	N/A	14	N/A	13
SF3B1	5	N/A	5	N/A	4
GNB1	6	N/A	6	N/A	5
NF1	1	1	N/A	0	1
Total	628	243	334	51	400

DP, depth; inf indel, in-frame insertion-deletion; N/A, not applicable variant consequence for this gene.

### Participant CHIP status

**Figure 3:**
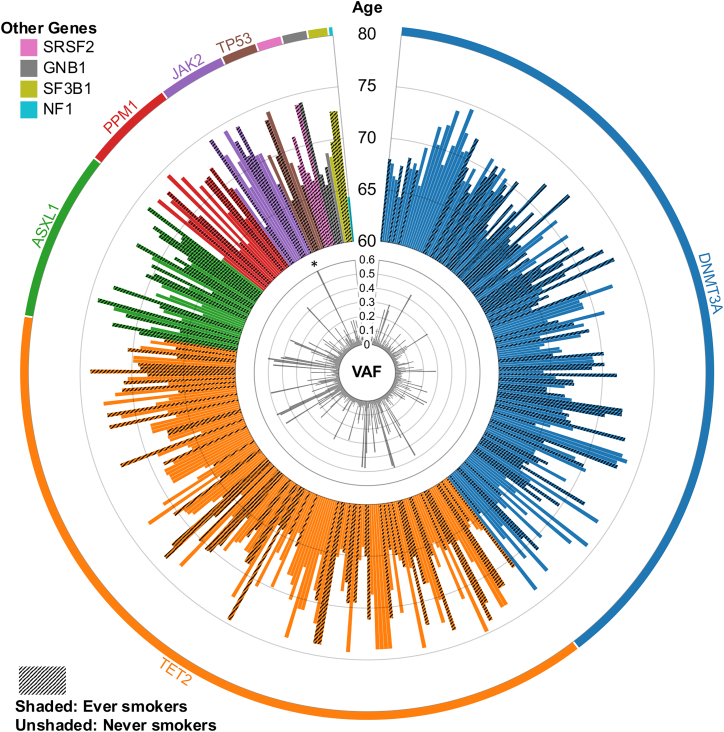
Rose chart of all CHIP-associated variants Each bar represents a different variant plotted against the age of the participant who had that variant. The VAF of the Mutect2 variant call is indicated in the center. This plot is based on plots from Xie et al.^14^ ∗This *JAK2* variant had a VAF of 0.76 and surpassed the boundary illustrated in this chart.

Three hundred and forty-seven participants (15%) were identified as CHIP positive with a total of 400 CHIP-associated variants (read depth ≥300) (Figure 3 and Table S3). Thirty-six of these variants were identified by at least one of the two calls to have a VAF >0.25. Forty-four individuals (2%) had multiple CHIP-associated variants, including 36 who had two variants, seven who had three variants, and one who had four variants (Figure S2). No somatic variation (within target regions) was identified in 1,442 (62%) who were considered CHIP negative. The remaining 539 (23%) had at least one somatic variant that could not be interpreted and were considered CHIP indeterminate.

The CHIP-indeterminate participants had a total of 999 variants with a VAF between 0.02 and 0.25. Of these variants, 736 (74%) were considered likely artifacts, mostly due to being identified by only one caller (*n* = 713, 97%). The remaining variants (*n* = 263, 26%) consisted of those that were missense (*n* = 88, 33%), synonymous (*n* = 59, 22%), splice dinucleotide (*n* = 40, 15%), UTR (*n* = 37, 14%), splice region not including the dinucleotide (*n* = 24, 9%), frameshift (*n* = 7, 3%), stop gained (*n* = 6, 2%), and in-frame indel (*n* = 2, 1%). Of these 263 variants, only four variants (2%) were considered CHIP associated according to the framework specified here (Table 2) but were identified with a read depth of <300.

### Associations with CHIP status

The proportion of individuals in each age tertile from lowest to highest who were CHIP positive was 12%, 16%, and 17%. The proportion of individuals in each age tertile who were CHIP indeterminate was 22%, 24%, and 23%. CHIP-positive participants were older than CHIP-indeterminate participants (*p* = 0.044), but no evidence of a difference in age distribution was observed between CHIP-indeterminate and CHIP-negative individuals (*p* = 0.150).

## Discussion

Large epidemiological and clinical studies are needed to develop clinical guidelines for the management of individuals with CHIP and their risk of disease. The generalizability of research depends on the method used to identify individuals with CHIP and those without. However, little guidance is available for the classification of variants as CHIP associated or how to identify individuals consistently and systematically as having CHIP. This study introduces a framework for categorizing individuals with CHIP, and those without, for downstream recruitment into epidemiological and clinical studies.

It was imperative that individuals with CHIP be identified with high confidence, but it was equally important to identify, with high confidence, those without CHIP. Individuals were considered CHIP positive if they had at least one genuine, somatic, CHIP-associated variant. However, determining an appropriate definition for the CHIP-negative group was more complicated. Grouping all individuals who were not CHIP positive together would introduce the possibility that some individuals carried a somatic variant that had insufficient evidence to assess whether it was CHIP associated or a true somatic variant that was mistakenly regarded as a likely artifact. Hence, individuals were only considered CHIP negative if they had no identified CHIP-associated variant (VAF ≥ 0.02) and no variants with a VAF between 0.02 and 0.25 in the exons or splice regions sequenced for the ten investigated genes. This required the definition of a third category, CHIP indeterminate, for individuals where it was not possible to confidently interpret the genetic variation observed from their panel testing data. This third category enabled the delineation of individuals who had uncertain molecular results from those confidently considered CHIP positive or CHIP negative. Hence, the framework developed here categorized individuals as either CHIP positive, negative, or indeterminate.

There were three independent elements to the execution of this framework, the first being whether the variant was genuine, the second whether the variant was somatic, and the third whether the variant was associated with CHIP. Assessing the authenticity of a variant presented a challenge because some were confidently considered either genuine variants or technical artifacts (recognized artifacts), while others were only suspected to be an artifact (likely artifacts). Recognized artifacts were excluded and comprised variants that were either seen too often to be genuine (>6% of samples^10^) or were sequencing run batch artifacts. In some instances, batch artifacts clustered in certain exons and generated doubt as to the technical validity of any other variants observed in these exons. These variants in regions with dubious sequencing quality were therefore considered likely artifacts. Other variants considered likely artifacts include those not identified by two independent variant callers or those that had a poor alignment profile when assessed in IGV. Likely artifacts were not excluded, and individuals with these could not be considered CHIP negative, due to the possibility that the suspected artifact was a true somatic variant and was CHIP associated. Neither recognized nor likely artifacts underwent variant curation.

The second element of this framework was to consider the likely origin of the variant as somatic or germline. Germline variants are expected to have a VAF greater than 0.25, and therefore it is reasonable to consider all variants with a VAF ≤0.25 as somatic. Some somatic variants induce large clonal proliferation and therefore may be seen with a VAF >0.25. However, not many individuals in this sample of participants with no reported personal history of cancer were expected to have a damaging CHIP-associated somatic variant at a VAF >0.25 in a myeloid malignancy gene. Hence, a variant with a VAF >0.25 was assumed to be germline unless it was a CHIP-associated variant. This is important, because a participant needed to have no somatic variation within the target regions to be considered CHIP negative. This was to limit the possibility of considering a participant CHIP negative who had a currently uninterpretable variant.

The third element of this framework was to determine whether variants were CHIP associated. Due to the large volume of genuine somatic variants, an expedient resource-mindful method of curating variants for CHIP association was required. Vlasschaert et al.,^7^ recently published a collated list of variants considered CHIP associated, and this was utilized here, with some adaptation, to appropriately and systematically assess variants. This list is a compilation of variants (or variant types) identified by the foundational CHIP epidemiology studies to be CHIP associated and comprised either rare truncating variants or recurrent variants detected in cancer genome datasets.^14^^,^^15^^,^^16^

This framework differs from those that have been published in two ways. The first is the method used for curating variants. Previous frameworks specify frameshift and splice-site variants as candidates for CHIP if they occur in *DNMT3A*, *TET2*, *ASXL1*, *PPM1D*, *TP53*, and *NF1*,^7^^,^^17^^,^^18^ but further guidance is needed for determining which frameshift and splice-site variants are likely to be deleterious. The framework here used evidence-based guidelines from germline and somatic variant curation frameworks^11^^,^^19^ to set an appropriate 3′ cutoff for frameshift and nonsense variants based on whether they are predicted to induce NMD, whether they impact a critical domain, or whether they introduce a PTC upstream of a different truncating variant that had been reported with germline disease for that gene. Regarding splice-site variants, this analysis was restricted to those that impact the splice dinucleotide, since these are most likely to have evidence of deleteriousness.^7^ Splice dinucleotide variants were only considered CHIP associated if they were curated by the genetics community (and reported in ClinVar) as pathogenic or likely pathogenic and had a spliceAI score of ≥0.20.^12^ Additionally, previous frameworks consider missense variants in *TET2* domains as candidates for CHIP, and these frameworks have specified that these domains occur at p.1104_1481 and p.1843_2002.^7^^,^^18^ However, based on the crystal structure of the protein,^13^ this framework recognized the position of the *TET2* domains as spanning p.1129_1481 and p.1843_1936.

Second, this framework differs in how it identifies CHIP-negative participants, and it defines a CHIP-indeterminate category. Previous frameworks utilize a dichotomy where individuals are either considered to have CHIP or not.^7^ However, it is possible that not all CHIP-associated variants have been identified by the research community, and such a dichotomy creates a scenario where some CHIP-negative individuals may have somatic variants where it is not clear if they are CHIP driver variants. Since CHIP-positive and -negative participants were to be recruited into a clinical study, the CHIP-indeterminate group was defined to categorize individuals who do not meet the stringent definitions for either of the two recruitment groups.

This study used the CHIP-indeterminate category as a means of managing participants with molecular data that could not be validated or interpreted. Clinical studies that determine CHIP status at participant recruitment will also need a strategy to manage complexities regarding uncertain molecular findings. Since a considerable number of likely artifacts (*n* = 736) were identified in CHIP-indeterminate participants, sequencing of a second sample using an orthogonal technology could help to determine the authenticity of variants. Additionally, a more individualized assessment of variants performed using established cancer variant curation frameworks^19^^,^^20^ may help to better categorize individuals as CHIP positive or CHIP negative.

Certain elements of this framework, including the variant curation methodology and the definition of CHIP negative, are generalizable and can be applied to studies that are of different size or sequencing design, whereas the most appropriate method for managing artifacts will vary based on the study design and sample size. Additionally, the definition of CHIP indeterminate was devised to consider circumstances where a somatic variant was identified at a VAF ≥0.02 in a myeloid malignancy gene but it is unclear whether the variant is deleterious. While this definition can be used by other studies, the heterogeneity in the CHIP-indeterminate group will be influenced by how variants are identified and interpreted.

This framework was successful in identifying 15% of participants in this dataset to have bona fide variants (VAF 0.02–0.76) associated with CHIP, with most variants occurring in *DNMT3A* and *TET2*. This finding is comparable to the prevalence of CHIP identified by previous research.^15^^,^^16^ Despite this sample being selected for a small age range (64–74 years), the occurrence of CHIP was associated with increased age, an established risk factor for CHIP. The age distribution of the CHIP-indeterminate participants more closely resembled the CHIP-negative participants, suggesting that the CHIP-indeterminate category is likely composed of more individuals without CHIP than those with CHIP.

Strengths of this study include the study design, large sample size, validated gene panel design, uniformity of sequencing technology, and the rigorous variant curation methodology. Additionally, a systematic identification and assessment of variants was performed, agreement of two variant callers was required, and the dataset was investigated for miscalled variants which, when identified, were assigned the correct call and reannotated. While many sequence variants could not be interpreted, a strength of this study was the definition of a CHIP-indeterminate group for individuals carrying these variants.

The limitations of this study include that the international clinical definition of CHIP was not strictly met.^4^^,^^21^ Specifically, data on the ABC Study participants to assess and exclude cases of cytopenia were not available, and we used DNA derived from saliva rather than blood or bone marrow. The impact of the latter is likely very small because recent reports demonstrated saliva to be an appropriate DNA source to detect CHIP-associated variants^5^ and observed the VAF detected in saliva-derived DNA to be highly concordant with the VAF detected in buffy-coat-derived DNA (*R*^2^ = 0.95).^6^ Further limitations include that the gene panel design and sequencing technology does not detect some types of genomic variation, including structural and large copy-number variants, and that not all protein-coding exons were targeted by the panel (Table S1). Also, the investigation was restricted to ten genes that were identified to have the most variants associated with CHIP in an investigation of whole-exome sequencing data from the UK Biobank.^7^ Several other genes that less frequently carry CHIP-associated variants (such as *GNAS*, *U2AF1*, and *CBL*) were not included in this study, and participants who carry CHIP-associated variants in these genes would not have been identified.

Future directions include the characterization of cardiovascular or cancer-related sequelae that may develop in this cohort. Risk models that predict the risk of CHIP progressing to a myeloid neoplasm are available,^2^^,^^3^ and now models need to be developed that predict the risk of a cardiovascular event for people with CHIP. By enabling clear categorization of CHIP-positive and -negative participants, future studies can be enriched with participants carrying somatic variants of interest to enable robust determination of the mechanisms underpinning enhanced cardiovascular risk, which would allow targeted treatment strategies to be developed.

In a rapidly evolving field, international approaches are needed to determine a clinically relevant definition of CHIP in terms of the genes mutated and the variant pathogenicity. While cancer variant curation guidelines are currently available,^19^^,^^20^ it is unclear whether these frameworks are appropriate for accurately capturing the clinical relevance of CHIP, which is associated with a range of non-malignant clinical conditions. Further development of standardized variant curation guidelines for CHIP will require the establishment of multidisciplinary expert panels with expertise in clinical genomics, functional genetics, and bioinformatics.

### Conclusions

This framework provides a practical resource-mindful method for identifying individuals with CHIP and those without for epidemiological, observational, and clinical research. Individuals were categorized as either CHIP positive, CHIP negative, or CHIP indeterminate. The CHIP-indeterminate group was defined to categorize individuals with uncertain molecular findings and differentiate them from those who carried no somatic variant in a CHIP-associated gene. The variant curation process adopted findings from previous investigations of CHIP, with sensible modifications that incorporated recognized knowledge of biological processes. Ultimately, this framework provides a consistent categorization of individuals as CHIP positive or CHIP negative and provides an opportunity for improved harmonization in the curation of CHIP.

## Data and code availability

All data associated with this report can be requested and obtained (after appropriate approval) via PEDIGREE (https://www.cancervic.org.au/research/epidemiology/pedigree).

## Acknowledgments

Australian Breakthrough Cancer (ABC) Study cohort recruitment was funded by Cancer Council Victoria and a generous gift from the Geary Estate. Ongoing data and sample collection and participant engagement is supported by Council Cancer Victoria. The collection of blood samples was supported by Gandel Philanthropy, as well as the Ian Potter Foundation, the Harry Secomb Foundation, and the Percy Baxter Charitable Trust, managed by Perpetual Trustees. This work was also funded by the Australian Medical Research Fund (principal investigator S.J.N.), the 10.13039/501100000925National Health and Medical Research Council (Investigator grant GNT2017325; M.C.S.), and the National Health Medical Research Council/Australian Medical Research Future Fund (Investigator grant GNT1195030, P.Y.). The ABC Study was conceptualized and established under the leadership of Professor Graham G. Giles, AM (PhD; Cancer Council Victoria, East Melbourne, VIC, Australia). We thank the ABC Study team, Helen Tsimiklis for sample management and preparation, and the tens of thousands of ABC Study participants from across Australia who generously contribute their time, data, and samples to make the study possible.

## Declaration of interests

The authors declare no competing interests.

## Web resources

BioRender, https://www.biorender.com
Ensemble-VEP (v.112) web interface, https://asia.ensembl.org/Multi/Tools/VEP?db=core
Online Mendelian Inheritance in Man (OMIM), https://www.omim.org

## Supplemental information

Document S1. Figures S1 and S2, Tables S1 and S2, and supplemental results

Table S3. List of identified CHIP-associated variants (*n* = 400)

Document S2. Article plus supplemental information

## References

[1] Singh J., Li N., Ashrafi E., Thao L.T.P., Curtis D.J., Wood E.M., McQuilten Z.K. (2024). Clonal hematopoiesis of indeterminate potential as a prognostic factor: a systematic review and meta-analysis. Blood Adv..

[2] Weeks L.D., Niroula A., Neuberg D., Wong W., Lindsley R.C., Luskin M., Berliner N., Stone R.M., DeAngelo D.J., Soiffer R. (2023). Prediction of risk for myeloid malignancy in clonal hematopoiesis. NEJM Evid..

[3] Gu M., Kovilakam S.C., Dunn W.G., Marando L., Barcena C., Mohorianu I., Smith A., Kar S.P., Fabre M.A., Gerstung M. (2023). Multiparameter prediction of myeloid neoplasia risk. Nat. Genet..

[4] Khoury J.D., Solary E., Abla O., Akkari Y., Alaggio R., Apperley J.F., Bejar R., Berti E., Busque L., Chan J.K.C. (2022). The 5th edition of the World Health Organization Classification of Haematolymphoid Tumours: Myeloid and Histiocytic/Dendritic Neoplasms. Leukemia.

[5] O’Reilly R.L., Burke J., Harraka P., Yeh P., Howlett K., Behrouzfar K., Rewse A., Tsimiklis H., Giles G.G., Bubb K.J. (2024). Saliva-derived DNA is suitable for the detection of clonal haematopoiesis of indeterminate potential. Sci. Rep..

[6] Stewart C.M., Parpart-Li S., White J.R., Patel M., Artz O., Foote M.B., Gedvilaite E., Lamendola-Essel M.F., Gerber D., Bhattacharya R. (2025). Clonal hematopoiesis detection by simultaneous assessment of peripheral blood mononuclear cells, blood plasma, and saliva. J. Clin. Investig..

[7] Vlasschaert C., Mack T., Heimlich J.B., Niroula A., Uddin M.M., Weinstock J., Sharber B., Silver A.J., Xu Y., Savona M. (2023). A practical approach to curate clonal hematopoiesis of indeterminate potential in human genetic datasets. Blood.

[8] O’Reilly R.L., Harraka P., Burke J., Belluoccio D., Yeh P., Howlett K., Behrouzfar K., Rewse A., Tsimiklis H., Giles G.G. (2025). Smart non-uniformity: Calibration of sequencing depth of a targeted gene panel to simultaneously detect somatic and germline variants. J. Mol. Diagn..

[9] Dharan N.J., Yeh P., Bloch M., Yeung M.M., Baker D., Guinto J., Roth N., Ftouni S., Ognenovska K., Smith D. (2021). HIV is associated with an increased risk of age-related clonal hematopoiesis among older adults. Nat. Med..

[10] Chan I.C.C., Panchot A., Schmidt E., McNulty S., Wiley B.J., Liu J., Turner K., Moukarzel L., Wong W.S.W., Tran D. (2024). ArCH: improving the performance of clonal hematopoiesis variant calling and interpretation. Bioinformatics.

[11] Abou Tayoun A.N., Pesaran T., DiStefano M.T., Oza A., Rehm H.L., Biesecker L.G., Harrison S.M., ClinGen Sequence Variant Interpretation Working Group ClinGen SVI (2018). Recommendations for interpreting the loss of function PVS1 ACMG/AMP variant criterion. Hum. Mutat..

[12] Walker L.C., Hoya M.d.l., Wiggins G.A.R., Lindy A., Vincent L.M., Parsons M.T., Canson D.M., Bis-Brewer D., Cass A., Tchourbanov A. (2023). Using the ACMG/AMP framework to capture evidence related to predicted and observed impact on splicing: Recommendations from the ClinGen SVI Splicing Subgroup. Am. J. Hum. Genet..

[13] Hu L., Li Z., Cheng J., Rao Q., Gong W., Liu M., Shi Y.G., Zhu J., Wang P., Xu Y. (2013). Crystal Structure of TET2-DNA Complex: Insight into TET-Mediated 5mC Oxidation. Cell.

[14] Xie M., Lu C., Wang J., McLellan M.D., Johnson K.J., Wendl M.C., McMichael J.F., Schmidt H.K., Yellapantula V., Miller C.A. (2014). Age-related mutations associated with clonal hematopoietic expansion and malignancies. Nat. Med..

[15] Jaiswal S., Fontanillas P., Flannick J., Manning A., Grauman P.V., Mar B.G., Lindsley R.C., Mermel C.H., Burtt N., Chavez A. (2014). Age-related clonal hematopoiesis associated with adverse outcomes. N. Engl. J. Med..

[16] Genovese G., Kähler A.K., Handsaker R.E., Lindberg J., Rose S.A., Bakhoum S.F., Chambert K., Mick E., Neale B.M., Fromer M. (2014). Clonal hematopoiesis and blood-cancer risk inferred from blood DNA sequence. N. Engl. J. Med..

[17] Niroula A., Sekar A., Murakami M.A., Trinder M., Agrawal M., Wong W.J., Bick A.G., Uddin M.M., Gibson C.J., Griffin G.K. (2021). Distinction of lymphoid and myeloid clonal hematopoiesis. Nat. Med..

[18] Jaiswal S., Natarajan P., Silver A.J., Gibson C.J., Bick A.G., Shvartz E., McConkey M., Gupta N., Gabriel S., Ardissino D. (2017). Clonal Hematopoiesis and Risk of Atherosclerotic Cardiovascular Disease. N. Engl. J. Med..

[19] Horak P., Griffith M., Danos A.M., Pitel B.A., Madhavan S., Liu X., Chow C., Williams H., Carmody L., Barrow-Laing L. (2022). Standards for the classification of pathogenicity of somatic variants in cancer (oncogenicity): Joint recommendations of Clinical Genome Resource (ClinGen), Cancer Genomics Consortium (CGC), and Variant Interpretation for Cancer Consortium (VICC). Genet. Med..

[20] Li M.M., Datto M., Duncavage E.J., Kulkarni S., Lindeman N.I., Roy S., Tsimberidou A.M., Vnencak-Jones C.L., Wolff D.J., Younes A., Nikiforova M.N. (2017). Standards and Guidelines for the Interpretation and Reporting of Sequence Variants in Cancer: A Joint Consensus Recommendation of the Association for Molecular Pathology, American Society of Clinical Oncology, and College of American Pathologists. J. Mol. Diagn..

[21] Arber D.A., Orazi A., Hasserjian R.P., Borowitz M.J., Calvo K.R., Kvasnicka H.M., Wang S.A., Bagg A., Barbui T., Branford S. (2022). International Consensus Classification of Myeloid Neoplasms and Acute Leukemias: integrating morphologic, clinical, and genomic data. Blood.

