# Supplementary material for: A framework for the molecular identification of CHIP for clinical research
Source: HGG Adv. 2026 Jan 20;7(2):100575. doi: 10.1016/j.xhgg.2026.100575 (PMC12925157; doi:10.1016/j.xhgg.2026.100575)
Supplement: Document S1. Figures S1 and S2, Tables S1 and S2, and supplemental results [file mmc1.pdf]

HGGA, Volume 7

Supplemental information

A framework for the molecular identification of CHIP for clinical research

Philip Harraka, Robert L. O'Reilly, Jared Burke, Paul Yeh, Kerryn Howlett, Kiarash Behrouzfar, Daniele Belluoccio, Amanda Rewse, Brigid M. Lynch, Kristen J. Bubb, Stephen J. Nicholls, Roger L. Milne, and Melissa C. Southey

Table of Contents

Supplemental Results 2

Figure S1. Circular lollipop plots of variants identified by gene 4

Figure S2. Rose chart of all gene co-mutations observed in 44 participants with multiple CHIP-associated variants 5

Table S1. Gene-specific exons targeted by panel sequencing 6

Table S2. Reannotation of incorrect variant calls 7

Table S3. List of identified CHIP-associated variants (n=400) 8

References 9

Supplemental Results

Artefacts

Recognised artefacts included recurrent variants observed with a VAF between 0.02 and 0.25 in more than 6% of sequenced samples (≥146/2425), and sequencing run batch artefacts. Nine recurrent variants were identified, and ten single nucleotide variants and four sets of indels were identified to be sequence run batch artefacts. There were multiple batch artefacts in exons 7 and 15 of *DNMT3A*, and exon 8 of *ASXL1*, and so all other variants in these exons or the flanking splice site dinucleotides were considered *likely artefacts*. Participants who had *likely artefacts* were considered CHIP-indeterminate (not CHIP-negative) because these could still be genuine CHIP-associated somatic variants.

Gene-specific variant findings

Variant findings for each gene are described under the respective heading.

DNMT3A (NM_022552.5)

DNMT3A was investigated for truncating, splice dinucleotide and missense (or inframe indel) variants. Truncating variants were classified as CHIP-associated if they introduced a PTC upstream of the last 50 bp of the penultimate exon (corresponding to a termination at, or upstream to, codon 849); the location at which NMD can no longer be assumed. This cut-off was considered appropriate because no reports of a truncating variant downstream of this location has been reported in an individual with a *DNMT3A* phenotype (ClinVar accessed June 2024). Two hundred and ninety-four variants were identified (Supplemental Figure 1A) in 276 participants. Of these, 162 variants were CHIP-associated (8 (5%) where at least one of the two calls reported a VAF >0.25) and 132 were not (11 (8%) with VAF >0.25). Five of the 162 CHIP-associated variants did not meet the read depth cut-off of ≥300, of which four (c.1229_1235del, p.Ala410GlyfsTer239; c.2357C>A, p.Ser786Ter; c.2368_2369insCA, p.Arg790ThrfsTer13; c.2446C>T, p.Gln816Ter) occurred in participants who were considered CHIP indeterminate, and the fifth (c.2477del, p.Lys826SerfsTer5) occurred in a person who was considered CHIP-positive because they carried another CHIP-associated variant that met the read depth cut-off. The total number of *DNMT3A* CHIP-associated variants with depth ≥300 was 157.

TET2 (NM_001127208.3)

TET2 was investigated for truncating/frameshift, splice dinucleotide and missense (or inframe indel) variants. Truncating variants were classified as CHIP-associated if they introduced a PTC upstream of (or at) amino acid p.Gly1936, which is the 3' end of the most C-terminal domain[1]. Missense variants were considered CHIP-associated if they were located within the minimal catalytically active fragment of TET2 (amino acids 1129-1481 and 1843-1936)[1]. Two hundred and twenty variants were identified (Supplemental Figure 1B) in 189 participants. Of these, 157 were CHIP-associated (21 (13%) where at least one of the two calls reported a VAF >0.25) and 63 were not (40 (63%) with VAF >0.25).

ASXL1 (NM_015338.6)

ASXL1 was investigated for truncating and splice dinucleotide variants. Truncating variants were classified as CHIP-associated if they were located in the last two exons of the transcript (exons 12 or 13) and introduced a PTC upstream of (or at) amino acid p.Arg1415, which is the location of the most 3' truncating variant reported with germline disease: c.4243C>T (p.Arg1415Ter)[2]. Thirty-three truncating variants were identified (Supplemental Figure 1C) in 33 participants. All variants occurred in the last 2 exons and were CHIP-associated (5 (15%) where at least one of the two calls reported a VAF >0.25). No splice dinucleotide variants were identified.

PPM1D (NM_003620.4)

PPM1D was investigated for truncating and splice dinucleotide variants. Truncating variants were classified as CHIP-associated if they were located in the last two exons of the transcript (exons 5 or 6) and introduced a PTC upstream of (or at) amino acid p.Arg552, which is the location of the most 3' truncating variant reported with germline disease: c.1654C>T (p.Arg552Ter)[3]. Twenty-two truncating variants were identified (Supplemental Figure 1D) in 22 participants. All variants occurred in the last exon, of which 18 were CHIP-associated (1 (6%) with VAF >0.25) and four were not (1 (25%) with VAF >0.25). No splice dinucleotide variants were identified.

TP53 (NM_000546.6)

TP53 was investigated for truncating, splice dinucleotide and missense (or inframe indel) variants. Truncating variants were classified as CHIP-associated if they introduced a PTC upstream of the last 50 bp of the penultimate exon (corresponding to a termination at, or upstream to, codon 350). Twenty variants were

identified (Supplemental Figure 1E) in 20 participants. Of these, seven were CHIP-associated (none with a VAF >0.25), and 13 were not (10 (77%) with VAF >0.25). No truncating variants were identified.

***SRSF2* (NM_001195427.2)**

SRSF2 was investigated for missense variants. Thirteen variants were identified in 13 participants. Of these, five were CHIP-associated (none with a VAF >0.25) including c.284C>T (p.Pro95Leu) in three participants, c.283C>A (p.Pro95Thr) in one, and c.284C>G (p.Pro95Arg) in one. Whereas eight missense variants were not CHIP-associated (3 (38%) with VAF >0.25) including c.170T>A (p.Phe57Tyr) in two participants and, in one participant each, c.337G>A (p.Gly113Ser), c.350G>A (p.Arg117His), c.538C>G (p.Arg180Gly), c.545G>A (p.Arg182Gln), c.577C>G (p.Pro193Ala) and c.614G>A (p.Arg205Gln).

***JAK2* (NM_004972.4)**

JAK2 exons 12 and 14 were investigated for missense variants. Fourteen variants were identified in 14 participants. Of these, 13 were the CHIP-associated variant c.1849G>T (p.Val617Phe) (one (8%) with VAF >0.25). Strikingly, one of these individuals had this variant at a VAF of 0.76 and had a haematological abnormality. The fourteenth variant, c.1599C>G (p.Asn533Lys), was not CHIP-associated and had a VAF >0.25.

***SF3B1* (NM_012433.4)**

SF3B1 exons 12 to 16 were investigated for missense variants. Five variants were identified in five participants. Of these, four were CHIP-associated (none with a VAF >0.25) including c.2098A>G (p.Lys700Glu) in three participants and c.1998G>T (p.Lys666Asn) in one. The fifth variant, c.2010G>T (p.Gln670His), was not CHIP-associated and did not have a VAF >0.25.

***GNB1* (NM_002074.5)**

GNB1 exon 5 was investigated for missense variants. Six variants were identified in six participants. Of these, five were CHIP-associated (none with a VAF >0.25) including c.169A>G (p.Lys57Glu) in four participants and c.170A>C (p.Lys57Thr) in one. The sixth variant, c.170A>G (p.Lys57Arg), was not CHIP-associated and did not have a VAF >0.25.

***NF1* (NM_001042492.3)**

NF1 exon 18 was investigated for truncating and splice dinucleotide variants. Truncating variants were classified as CHIP-associated if they introduced a PTC upstream of the last 50 bp of the penultimate exon (corresponding to a termination at, or upstream to, codon 2776). One variant was identified, c.2033dup (p.Ile679AspfsTer21), that was CHIP-associated and did not have a VAF >0.25. No splice dinucleotide variants were identified.

Gene co-mutation

Forty-four individuals (2%) had multiple CHIP-associated variants (Supplemental Figure 2), of which 30 had variants in at least two different genes. Fourteen participants had a combination of *DNMT3A* and *TET2* CHIP, five had a combination of *TET2* and *ASXL1* CHIP, and two had a combination of *TET2* and *JAK2* CHIP. The remaining nine individuals had the following combinations: *ASXL1*/*SF3B1*, *DNMT3A*/*JAK2*, *DNMT3A*/*TET2*/*SF3B1*, *DNMT3A*/*TP53*, *TET2*/*JAK2*/*PPM1D*, *TET2*/*PPM1D*, *TET2*/*SF3B1*, *TET2*/*SRSF2* and *TET2*/*TP53*.

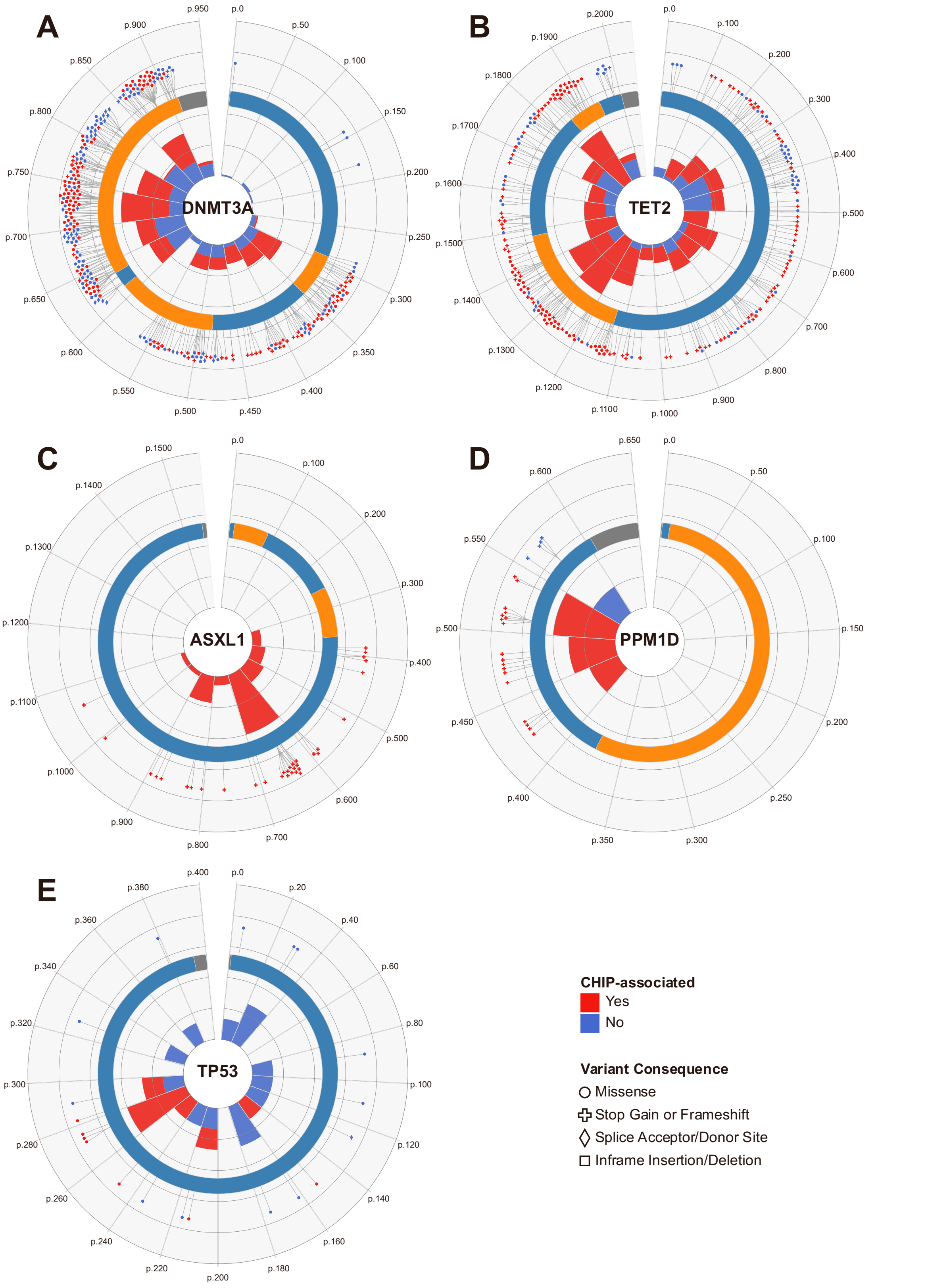

Figure S1. Circular lollipop plots of variants identified by gene (at any depth). Inner and outermost segments of the plots are separated by a blue bar that represents the length of the protein, ending at the beginning of the grey bar, and overlayed by orange bars that represent UniProt domains (accessed 25/09/2024). The lollipop (outer) segment of the graph plots the identified variants by the location of their impact on the protein (or, for splice acceptor or donor site variants, the nearest amino acid), whether they are CHIP-associated (red) or not (blue), and the variant consequence. The bar graph (inner segment) indicates the relative distribution of all gene variants according to the location of their impact on the protein, and the proportion of variants that are CHIP-associated. Plots indicate distribution of (**A**) *DNMT3A* variants (UniProt PDB Q9Y6K1), (**B**) *TET2* variants (UniProt PDB Q6N021), (**C**) *ASXL1* variants (UniProt PDB Q8IXJ9), (**D**) *PPM1D* variants (UniProt PDB O15297) and (**E**) *TP53* variants (UniProt PDB P04637). Missense variants in *ASXL1* and *PPM1D* were not investigated or reported.

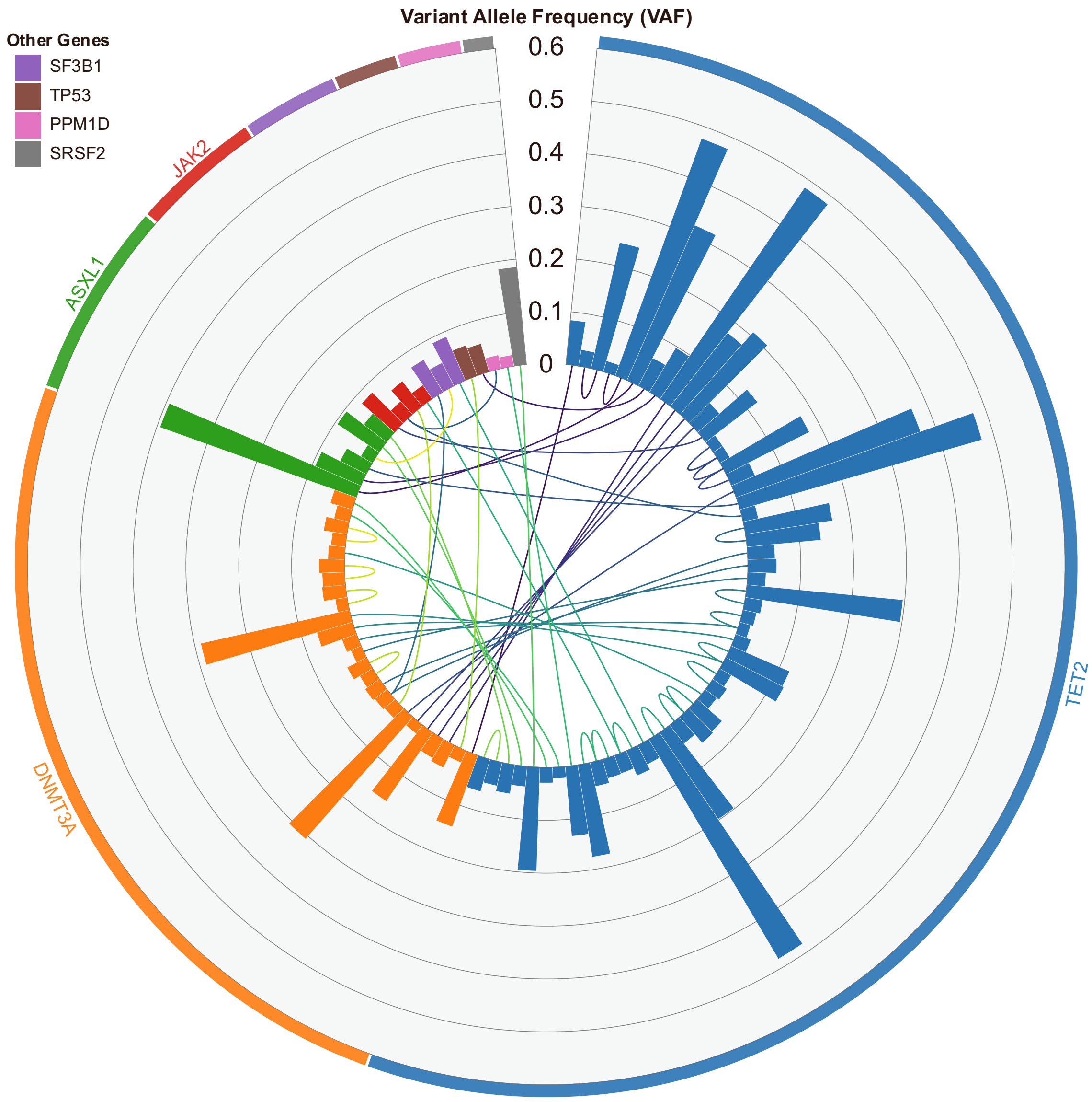

Figure S2. Rose chart of all gene co-mutations observed in 44 participants with multiple CHIP-associated variants. Each bar represents a different variant plotted against the VAF. Variants identified in the same participant are linked by a line in the centre.

Table S1. Gene-specific exons targeted by panel sequencing

Gene	Refseq transcript	Exons targeted
DNMT3A	NM_022552.5	2-23
TET2	NM_001127208.3	3-11
ASXL1	NM_015338.6	All exons
PPM1D	NM_003620.4	All exons
TP53	NM_000546.6	2-11
SRSF2	NM_001195427.2	1, 2
JAK2	NM_004972.4	12, 14
SF3B1	NM_012433.4	12-16
GNB1	NM_002074.5	5
NF1	NM_001042492.3	18

Table S2. Reannotation of incorrect variant calls

Genomic coordinates (GRCh38) of variant call	Gene	HGVS c.* of variant call	Observed variant from alignment	HGVS c.* of observed variant[†]
**Mutect2**	*TET2*		4:105272591 CGAGA> CAAT	4:105272592-GAGA>AAT c.4211_4214delinsAAT p.Arg1404GlnfsTer44
4:105272591 CGAG>C		c.4211_4213del		
4:105272595 A>AAT		c.4215_4216dup		
**Vardict**				
4:105272592 G>A		c.4211G>A		
**Mutect2**	*ASXL1*[‡]		20:32435359 GAAAACT> GAAAC	20:32435363-ACT>C c.2651_2653delinsC p.Asn884ThrfsTer9
20:32435359 GA>G		c.2651del		
20:32435364 CT>C		c.2654del		
**Mutect2**	*ASXL1*		20:32435333 CTAT>CAA	20:32435334-TAT>AA c.2622_2624delinsAA p.Met875ArgfsTer11
20:32435333 CT>C		c.2622del		
20:32435336 T>A		c.2624T>A		
**Vardict**				
20:32435334 T>A		c.2622T>A		
20:32435335 AT>A		c.2624del		

*Variant nomenclature according to the Human Genome Variation Society (HGVS) coding DNA sequence.
[†]Ensemble-VEP (v112) web interface used for reannotation (accessed 01 Aug 2024; https://asia.ensembl.org/Multi/Tools/VEP?db=core).
[‡]At the genomic coordinates of this variant, VarDict reported a multiallelic site but did not call this variant. The observed alignment (IGV) profile for this variant is satisfactory and the Mutect2 VAF was 0.19.

Table S3. List of identified CHIP-associated variants (N=400)

The chromosome (chrom), position number (pos), and reference (ref) and alternate (alt) nucleotide sequences are specified according to VCF standards with genomic coordinates given according to the GRCh38 human reference genome. The gene where the variant was found is indicated by the column vep_symbol. Variant nomenclature is according to the Human Genome Variation Society (HGVS) for the coding DNA sequence (vep_hgvsc) and protein sequence (vep_hgvsp). The variant allele fraction (vaf) and depth (dp) for the Mutect2 and VarDict calls are specified for each variant. All single nucleotide variants were annotated with the “oncogenic” field from the oncoKB database[4,5] which was queried on the 19th of November 2025. All variants were annotated with the ClinVar “classification” and “review_status” fields (dated 16-November-2025). A comment is provided for variants that were reannotated (notes) as per Table S2.

References

1. Hu, L., Li, Z., Cheng, J., Rao, Q., Gong, W., Liu, M., Shi, Y.G., Zhu, J., Wang, P., and Xu, Y. (2013). Crystal Structure of TET2-DNA Complex: Insight into TET-Mediated 5mC Oxidation. Cell *155*, 1545–1555. https://doi.org/10.1016/j.cell.2013.11.020.
2. Negri, G., Magini, P., Milani, D., Crippa, M., Biamino, E., Piccione, M., Sotgiu, S., Perrìa, C., Vitiello, G., Frontali, M., et al. (2019). Exploring by whole exome sequencing patients with initial diagnosis of Rubinstein-Taybi syndrome: the interconnections of epigenetic machinery disorders. Hum Genet *138*, 257–269. https://doi.org/10.1007/s00439-019-01985-y.
3. Jansen, S., Geuer, S., Pfundt, R., Brough, R., Ghongane, P., Herkert, J.C., Marco, E.J., Willemsen, M.H., Kleefstra, T., Hannibal, M., et al. (2017). De Novo Truncating Mutations in the Last and Penultimate Exons of PPM1D Cause an Intellectual Disability Syndrome. Am J Hum Genet *100*, 650–658. https://doi.org/10.1016/j.ajhg.2017.02.005.
4. Suehnholz, S.P., Nissan, M.H., Zhang, H., Kundra, R., Nandakumar, S., Lu, C., Carrero, S., Dhaneshwar, A., Fernandez, N., Xu, B.W., et al. (2024). Quantifying the Expanding Landscape of Clinical Actionability for Patients with Cancer. Cancer Discov. *14*, 49–65. https://doi.org/10.1158/2159-8290.CD-23-0467.
5. Chakravarty, D., Gao, J., Phillips, S.M., Kundra, R., Zhang, H., Wang, J., Rudolph, J.E., Yaeger, R., Soumerai, T., Nissan, M.H., et al. (2017). OncoKB: A Precision Oncology Knowledge Base. JCO Precis Oncol *2017*. https://doi.org/10.1200/po.17.00011.
